# Combined use of subclinical hydroxyurea and CHK1 inhibitor effectively controls melanoma and lung cancer progression, with reduced normal tissue toxicity compared to gemcitabine

**DOI:** 10.1002/1878-0261.12497

**Published:** 2019-06-14

**Authors:** Zay Yar Oo, Martina Proctor, Alexander J. Stevenson, Deborah Nazareth, Madushan Fernando, Sheena M. Daignault, Catherine Lanagan, Sebastian Walpole, Vanessa Bonazzi, Dubravka Škalamera, Cameron Snell, Nikolas K. Haass, Jill E. Larsen, Brian Gabrielli

**Affiliations:** ^1^ Smiling for Smiddy Research Group Translational Research Institute Mater Research Institute‐The University of Queensland Brisbane Australia; ^2^ Translational Research Institute The University of Queensland-Diamantina Institute Brisbane Australia; ^3^ Translational Research Institute Queensland University of Technology Brisbane Australia; ^4^ Mater Pathology Mater Adults Hospital Mater Misericordiae Limited South Brisbane Australia; ^5^ QIMR-Berghofer Medical Research Institute The University of Queensland Brisbane Australia; ^6^ School of Medicine The University of Queensland Brisbane Australia

**Keywords:** CHK1 inhibitor, hydroxyurea, macrophage infiltration, replication stress

## Abstract

Drugs such as gemcitabine that increase replication stress are effective chemotherapeutics in a range of cancer settings. These drugs effectively block replication and promote DNA damage, triggering a cell cycle checkpoint response through the ATR–CHK1 pathway. Inhibiting this signalling pathway sensitises cells to killing by replication stress‐inducing drugs. Here, we investigated the effect of low‐level replication stress induced by low concentrations (> 0.2 mm) of the reversible ribonucleotide reductase inhibitor hydroxyurea (HU), which slows S‐phase progression but has little effect on cell viability or proliferation. We demonstrate that HU effectively synergises with CHK1, but not ATR inhibition, in > 70% of melanoma and non‐small‐cell lung cancer cells assessed, resulting in apoptosis and complete loss of proliferative potential *in vitro* and *in vivo*. Normal fibroblasts and haemopoietic cells retain viability and proliferative potential following exposure to CHK1 inhibitor plus low doses of HU, but normal cells exposed to CHK1 inhibitor combined with submicromolar concentrations of gemcitabine exhibited complete loss of proliferative potential. The effects of gemcitabine on normal tissue correlate with irreversible ATR–CHK1 pathway activation, whereas low doses of HU reversibly activate CHK1 independently of ATR. The combined use of CHK1 inhibitor and subclinical HU also triggered an inflammatory response involving the recruitment of macrophages *in vivo*. These data indicate that combining CHK1 inhibitor with subclinical HU is superior to combination with gemcitabine, as it provides equal anticancer efficacy but with reduced normal tissue toxicity. These data suggest a significant proportion of melanoma and lung cancer patients could benefit from treatment with this drug combination.

**Abbreviations**
ATR: ataxia telangiectasia and RAD3‐related
CHK1: checkpoint kinase 1
CHK1i: CHK1 inhibitors
EdU: ethynyl‐2′‐deoxyuridine
HU: hydroxyurea
NSCLC: non‐small‐cell lung cancer
RNR: ribonucleotide reductase
TS: tumour sphere

## 1 Introduction

Replication stress is a common feature of cancers, produced by excessive mitogenic signalling and high levels of DNA damage (Gaillard *et al*., 2015). Inhibitors of replication such as bulky DNA adducts from UV damage or cigarette smoke, and inhibitors of DNA polymerase or ribonucleotide reductase (RNR) activity such as hydroxyurea (HU) and gemcitabine promote replication stress by slowing or blocking replication fork progression. In the latter case, an S‐phase cell cycle checkpoint response is triggered, caused by helicase unwinding of the double‐stranded DNA exceeding the progression of the replication fork. This results in extended regions of single‐stranded DNA that is recognised by the RPA complex which recruits ataxia telangiectasia and RAD3‐related (ATR) and in turn activates checkpoint signalling via checkpoint kinase 1 (CHK1) to block origin firing and S‐phase progression until the stress is relieved (Dobbelstein and Sorensen, 2015; Gaillard *et al*., 2015). Double‐stranded DNA breaks that can occur as a consequence of replication fork collapse are repaired by homologous recombination (HR) repair, and this is also regulated by CHK1 (Zuazua‐Villar *et al*., 2015).

The relatively small proportion of tumours with high levels of endogenous replication stress can be targeted by CHK1 inhibitors (CHK1i) as a single agent to selectively destroy these tumours (Brooks *et al*., 2013; Oo *et al*., 2018; Sakurikar *et al*., 2016). CHK1i also synergise potently with replication stress inducers *in vitro* (Xiao *et al*., 2013). In the clinic, CHK1i are primarily being investigated as chemosensitising agents, and to date, there have been > 15 trials of various CHK1i in combination primarily with gemcitabine (McNeely *et al*., 2014). Recent clinical trials using several different CHK1i have shown that the combination with gemcitabine has clinical activity, and although there were severe haematological adverse events, they were reported to be clinically manageable (Calvo *et al*., 2016; Daud *et al*., 2015; Italiano *et al*., 2018). These combinations utilise doses of gemcitabine that alone completely blocks S‐phase progression and triggers ATR–CHK1 activation. Inhibiting CHK1 or ATR in this context is equivalent, driving both replication catastrophe and apoptosis (Buisson *et al*., 2015).

Here, we have investigated the effects of triggering low levels of replication stress *in vivo* using low doses of HU. Low‐level replication stress imposed by low concentrations of HU (0.2 mm) or low oxygen tensions (2% O_2_) is permissive for replication, albeit at reduced rates (Alver *et al*., 2014), and, unlike high concentrations of HU (1–2 mm), has only a minor effect on dNTP pools (Techer *et al*., 2016). This low‐level replication stress results in CHK1 activation, reportedly via DNA‐PK rather than ATR (Buisson *et al*., 2015), which regulates origin firing by blocking late origins but allowing dormant origins within active replication factories to initiate replication (Ge and Blow, 2010). The consequence of CHK1 inhibition in conditions of replication stress is a massive increase in the numbers of origins fired, which can lead to RPA exhaustion, exposure of ssDNA and DNA strand breaks (Toledo *et al*., 2013). HU is still used as a chemotherapeutic agent in myeloproliferative neoplasms (Ahn *et al*., 2013) and sickle‐cell disease (Wiczling *et al*., 2014). It is well tolerated with limited toxicities. The low concentrations of HU required for synergistic effects with CHK1i are readily achieved with normal clinical doses used (20–30 mg·kg^−1^), and well below the doses used clinically (Wiczling *et al*., 2014). We demonstrate melanomas and non‐small‐cell lung carcinomas (NSCLC), which are both reported to have high levels of replication stress (Oo *et al*., 2018; Syljuasen *et al*., 2015), are sensitive to the combination of subclinical‐dose HU with the CHK1i GDC‐0575 *in vitro* and *in vivo*. This combination is well tolerated and causes minimal haematological effects *in vivo*. Normal cells can recover from this combination, whereas they completely lose proliferative capacity with a similar combination with gemcitabine.

## 2 Materials and methods

### 2.1 Cell lines

The panel of melanoma tumour sphere (TS) lines was developed and cultured as described previously (Oo *et al*., 2018). The NSCLC cell lines Calu‐1, H1299, H1650, H1792, H1975, H2052, H322, H358, H82, HCC2429 and HCC515 were cultured in RPMI‐1640 medium (Sigma‐Aldrich, St Louis, MO, USA; R0883) containing 10% FBS (Bovogen, Victoria, Australia), 2.5 mm HEPES (Sigma‐Aldrich), 1 mm sodium pyruvate (Life Technologies, Carlsbad, CA, USA) and 2 mm l‐glutamine (Life Technologies). NFF cells were cultured in DMEM (Sigma‐Aldrich; D5671) containing 10% FBS (Bovogen), 2.5 mm HEPES (Sigma‐Aldrich), 1 mm sodium pyruvate (Life Technologies) and 2 mm l‐glutamine (Life Technologies). Human melanoblast lines (QF1597, QF1610, QF1618, QF1619) were derived from individual donors and cultured with MCDB medium as described in Cook *et al*. (2003). Primary human adult melanocytes (HEMa; Life Technologies) were cultured with Medium 254 supplemented with HMGS‐2 (Life Technologies). The identity of all cell lines was confirmed by STR fingerprinting and used within 3 months of thawing. All cell lines were confirmed to be mycoplasma free. All TS and NSCLC lines were cultured in 2% O_2_ and 5% CO_2_ at 37 °C in a Binder low‐O_2_ incubator, as this is a more physiological O_2_ level. CHK1 inhibitors GNE‐323 and GDC‐0575 were provided by Genentech, South San Francisco, CA, USA.

Dose–response experiments were performed as described previously (Brooks *et al*., 2013). For TS dose–response experiments, cells were seeded and allowed to form spheres for 24 h before drug treatment for 72 h. Cell viability for TS cultures was determined by CellTiter‐Glo 3D cell viability assay in triplicate. Cell viability of NSCLC lines was determined using the resazurin assay. IC_50_ values (concentration required to reduce viability by 50%) were calculated for each of the cell lines. The data are the mean and SD of triplicate determinations. Flow cytometry for DNA content was performed as described previously (Oo *et al*., 2018). Cells were labelled with 10 μm ethynyl‐2′‐deoxyuridine (EdU) in complete media for 2 h and then harvested and the cells fixed with 4% formaldehyde. EdU was labelled using CLICK chemistry with Alexa488‐azide and DNA with 4′,6‐diamidino‐2‐phenylindole (DAPI), and then, the percentage of EdU‐positive cells was quantified using high‐content imaging as described in Oo *et al*. (2018).

### 2.2 Immunoblotting

Cell pellets were lysed and prepared for immunoblotting as previously described (Brooks *et al*., 2014). Lysates were equalised for protein content and then separated by SDS/PAGE and transferred to PVDF membranes. Membranes were probed with antibodies against cleaved PARP, cleaved caspase‐3, γH2AX, pCHK1 Ser317, BCL‐2, BCL‐XL, BIM, BAD, BID (Cell Signaling Technology, Danvers, MA, USA), RPA2, RRM2, MCL1 (Santa Cruz Biotechnology, Dallas, TX, USA), pRPA2 Ser33, pRPA2 Ser4/8 (Bethyl Laboratories, Montgomerey, TX, USA), NOXA (Abcam, Cambridge, UK), PUMA (Calbiochem, San Diego, CA, USA) and α‐tubulin (Sigma‐Aldrich). Proteins were visualised using chemiluminescence detection.

### 2.3 MCL1 overexpression

MCL1 ORF was sequence‐verified and cloned into pLEX 307. Lentiviral supernatant for transduction of cells was produced as previously described (Skalamera *et al*., 2012), and melanoma cell lines were transduced as previously described (Skalamera *et al*., 2016).

### 2.4 EdU labelling

For EdU staining, cells were treated with or without 0.1 mm HU for 24 h and then labelled for 2 h with EdU prior to fixation and staining of incorporated EdU as described in Ranall *et al*. (2010). Plates were scanned on an InCell 2000 (GE, Boston, MA, USA), images were then analysed using the cellprofiler analysis software (Jones *et al*., 2008), and data were processed in rstudio (https://cran.r-project.org/).

### 2.5 Xenograft studies

All animal experiments were performed according to the guidelines of the Australian and New Zealand Council for the Care and Use of Animals in Research and approved by the University of Queensland Animal Ethics number UQDI/049/14/CA. Four‐ to six‐week‐old female nude BALB/c mice were injected with 1–2 × 10^6^ cells in Matrigel (BD Biosciences, San Jose, CA, USA) by subcutaneous injection on the hind flank. For each treatment arm, 4–6 mice were used. Once tumours reached approximately 100 mm^3^, mice were treated with CHK1i, HU or vehicle (0.5% w/v methylcellulose and 0.2% v/v Tween‐80) by oral gavage, then HU ip for three cycles where one cycle comprised treatment on three alternative days a week. Tumour size was measured three times per week using callipers. Mice were killed at up to 6 weeks after terminating the treatment or when tumour size measured > 1 cm^3^. Immunohistochemical staining for γH2AX (Cell Signaling), Ki67 (Dako, Glostrup, Denmark), HMGB1 (Sigma‐Aldrich) and F4/80 (Novus Biologicals, Centennial, CO, USA) was as described previously (Oo *et al*., 2018). Statistical analysis was performed by either two‐tailed *t*‐test for comparison of two conditions or two‐way ANOVA for tumour growth using graphpad prism 7, GraphPad Software, San Diego, CA, USA.

## 3 Results

### 3.1 Subclinical concentrations of HU slow S‐phase progression and sensitise to CHK1i

CHK1i have been shown to strongly synergise with drugs that promote replication stress, particularly the RNR inhibitors gemcitabine and HU (Montano *et al*., 2012; Xiao *et al*., 2013). The combination with gemcitabine has been trialled in patients with limited success, with dose‐limiting toxicities being a major shortcoming of this combination (Daud *et al*., 2015; Infante *et al*., 2017). Here, we have investigated the effects of subclinical concentrations of gemcitabine and HU in NSCLC and melanoma cell lines and TS cultures, and in normal skin fibroblasts to compare the effects of these RNR inhibitors on synergy with the CHK1i GDC‐0575 (Oo *et al*., 2018).

**Figure 1:**
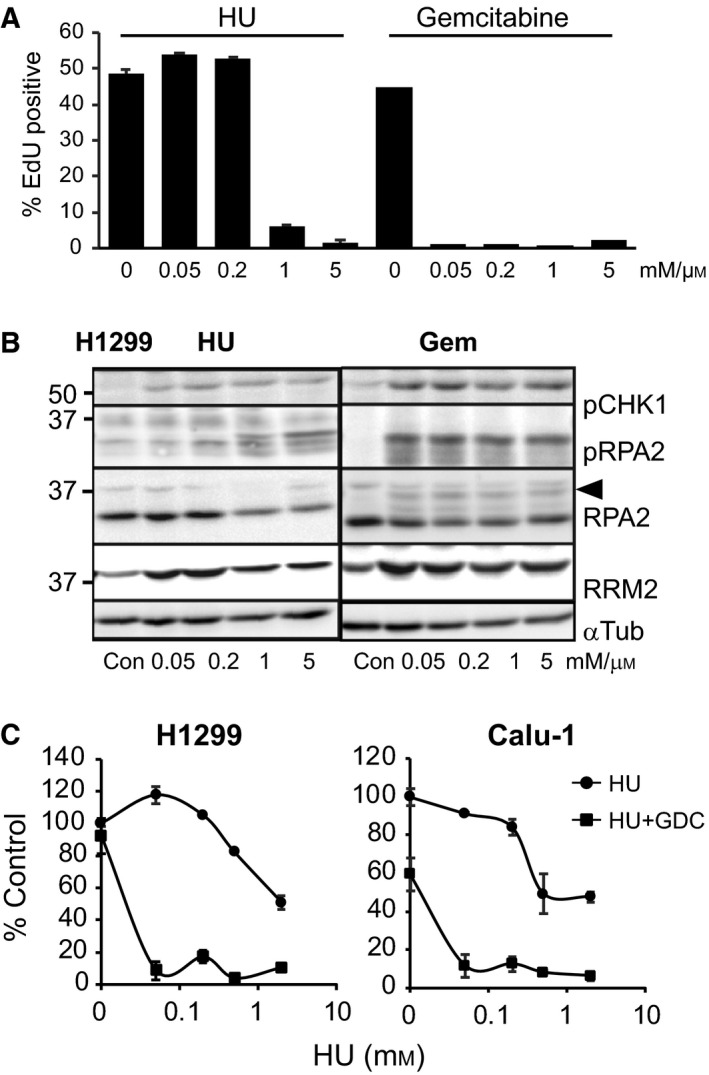
Low‐dose HU strongly sensitises tumour cells to CHK1i. (A) The NSCLC cell line H1299 was treated with the indicated concentrations of HU or gemcitabine (Gem) for 24 h and then harvested and analysed for S‐phase arrest using EdU incorporation measured by high‐content imaging. (B) H1299 cells treated as described in A were lysed and immunoblotted for the indicated markers of replication stress. The hyperphosphorylated form of RPA2 is indicated by the arrowhead. α‐Tubulin is a loading control. (C) Dose response of H1299 and Calu‐1 NSCLC cell lines with and without 0.5 μm GDC‐0575. Cells were treated for 72 h, and then, viability was assayed using resazurin. The data are expressed as percentage of the untreated control. The error bars represent standard deviation.

A dose response comparing gemcitabine and HU in two NSCLC cell lines, H1299 and Calu‐1, showed that nanomolar doses of gemcitabine resulted in S‐phase arrest, indicated by the loss of EdU incorporation. By comparison, sub‐mm concentrations of HU had little effect whereas mm concentrations of HU blocked S phase as effectively as gemcitabine (Fig. 1A, Fig. S1A). Phosphorylation of the activating Ser317 on CHK1 (pCHK1) and increased levels of RRM2, a product of CHK1 activation (Zhang *et al*., 2009), were also observed from the lowest concentration of gemcitabine and HU (Fig. 1B, Fig. S1B). High levels of replication stress were similar across all concentrations of gemcitabine, indicated by high levels of phosphorylation of replication protein A2 (RPA2) S33, a target of ATR (Liu *et al*., 2012); and hyperphosphorylation of RPA2, illustrated in the RPA2 immunoblot by increased level of the 37‐kDa band and reduced level of the 32‐kDa band, whereas the lower concentrations of HU produced comparatively little RPA2 S33 phosphorylation and hyperphosphorylation (Fig. 1B; Fig. S1B). Concentrations up to 0.2 mm HU had little effect on the viability or proliferation of NSCLC cells, whereas 0.05 mm HU maximally sensitised both cell lines to 0.5 μm GDC‐0575 (Fig. 1C). The surviving cells had completely lost their proliferative potential, evidenced by a lack of colony‐forming potential after washout of the combination treatment, with little difference between 0.1 mm HU and 2 mm HU + GDC‐0575 combination treatments (Fig. S2A).

**Figure 2:**
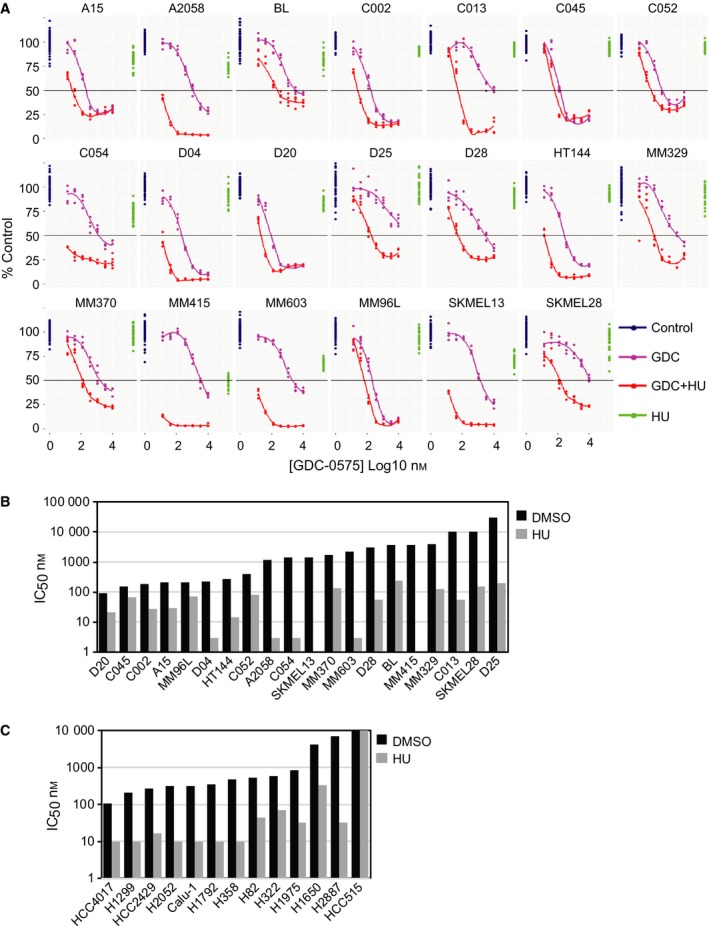
Melanoma and NSCLC lines are sensitised to CHK1i with low‐dose HU. (A) Dose response (in log10) of the indicated melanoma TS lines with and without 0.2 mm HU and the CHK1i GDC‐0575. 50% viability is indicated by the horizontal line. (B, C) IC_50_ values for GDC‐0575 in the panel of melanoma TS (B) and NSCLC cell lines (C), with and without 0.2 mm HU. The drugs were added together, and cell viability was assayed after 72‐h drug treatment. The melanoma data are the mean of two separate determinations.

The ability of a subclinical concentration of HU (0.2 mm) to sensitise melanoma TS cultures and NSCLC cell lines (attached cultures) to GDC‐0575 was assessed. Subclinical HU reduced viability by > 20% in only three of 20 melanoma TS (Fig. 2A). Melanoma TS previously reported to be sensitive to CHK1i as a single agent (A15, C002, C045, D20, MM96L, Oo *et al*., 2018) showed little increase in sensitivity to GDC‐0575 when combined with HU. Another 14 TS lines showed a ≥ 10‐fold decrease in IC_50_ values (Fig. 2A,B; Table S1A). The combination treatment produced a complete loss of viability in some TS lines (e.g. A2058, D04, MM415), while others showed a reduction in viability to 20–30% of controls (e.g. D20, C002; Fig. 2A). These surviving cells did not have any proliferative capacity, demonstrated by the lack of colony‐forming ability in adherent culture after removal of the combination treatment (0.5 μm GDC‐0575 + 0.2 mm HU, 3 days; Fig. S2B). This concentration of GDC‐0575 was used for the remaining experiments and was within a range readily attained in patients (Italiano *et al*., 2018). Interestingly, the ability of subclinical HU to sensitise to CHK1i was not replicated with the ATR inhibitor VE‐821, where sensitisation was only observed at millimolar doses of HU (Fig. S3).

In the 13 NSCLC cell lines assessed, four had a 50% reduction in viability when treated with HU alone and half of the lines tested had up to 20% reduction in viability (Fig. S4). All the NSCLC cell lines showed a ≥ 8‐fold decrease in IC_50_ values in the combined treatment, except HCC515, which was resistant across all doses examined (Fig. 2C; Fig. S4, Table S1B).

### 3.2 CHK1i combination with HU but not gemcitabine is reversible in normal cells

**Figure 3:**
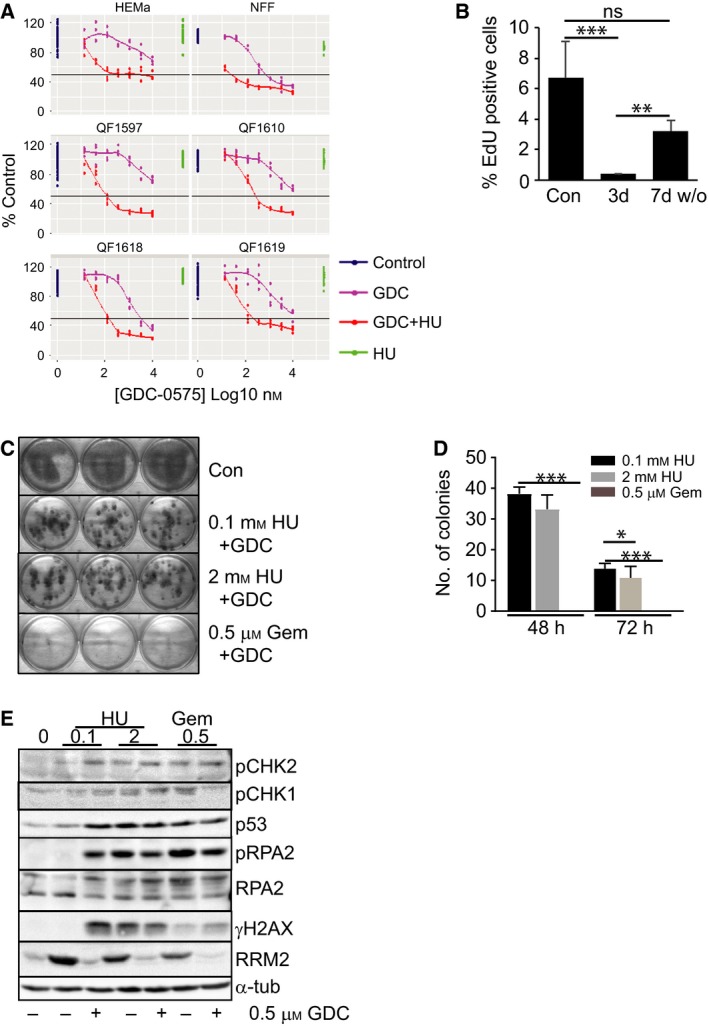
Normal cells recover from HU + GDC‐0575 treatment. (A) Dose response of primary adult melanocyte (HEMa), foreskin fibroblast (NFF) and neonatal melanoblasts (QF series) with and without 0.2 mm HU and the CHK1i GDC‐0575. The drugs were added together. 50% viability is indicated by the horizontal line. The data for each replicate are shown. (B) Flow cytometric analysis of EdU incorporation into NFF cells (% EdU positive cells). Results from NFF cells without drugs (Con) are shown compared to 0.2 mm HU + 0.5 μm GDC‐0575 treated cells for three days (3 d) and 0.2 mm HU + 0.5 μm GDC‐0575 treated for three days, cells washed free of drug and grown for a further 7 days (7 d w/o). The data show the mean and SD of triplicate assays. (C) NFFs were treated with and without the indicated concentrations of HU and gemcitabine + 0.5 μm GDC‐0575 for 48 h, and then, the drug was washed out and replaced with fresh media. Cells were allowed to grow for another 10 days, except controls which were fixed and stained with crystal violet after 4 days. (D) Quantification of the number of colonies from the experiment shown in C and a parallel experiment where the drug treatment was washed out after 3 days. The data are from six replicate wells. No colonies were found with gemcitabine treatment. (E) Immunoblot of the indicated proteins and markers of DNA damage and replication stress after 48 h of the indicated treatments. These are representative of replicate experiments. Significance was assessed by two‐tailed *t*‐test. *P < 0.05; **P < 0.005; ***P < 0.0001. ns, not significant. The error bars represent standard deviation.

Six primary normal cell lines, foreskin fibroblasts (NFF), adult melanocytes (HEMa) and four neonatal melanoblast lines from different donors (QF series) were assessed for their sensitivity to single‐agent GDC‐0575 and the ability of 0.2 mm HU to sensitise to the CHK1i. The normal cell lines displayed increasing loss of viability at concentrations of single‐agent GDC‐0575 above 0.1 μm, NFF being the most sensitive, but none showed any loss of viability with 0.2 mm HU as a single agent. All lines were sensitised to GDC‐0575 with 0.2 mm HU, although all had > 25% viable cells at even the highest concentration of GDC‐0575 used (Fig. 3A). Treatment of NFF cultures, the most sensitive to the combination, with 0.2 mm HU + 0.5 μm GDC‐0575 completely blocked DNA replication indicated by the loss of EdU‐positive cells after 3 days of treatment, but cells recovered after removal of the drug, with no significant difference in the proportion of EdU‐positive cells by 7 days after drug removal from the untreated controls (Fig. 3B). To assess whether the arrested normal fibroblasts from the drug combination treatment retained long‐term proliferation potential, fibroblasts were treated for 2 or 3 days with 0.5 μm GDC‐0575 in combination with 0.1 or 2 mm HU or 0.5 μm gemcitabine and then washed and fresh medium added. We observed strikingly different effects on proliferative capability. All drug combinations resulted in growth inhibition compared with controls, which reached confluency and were fixed and stained 7 days before treating cultures. Surprisingly, the 2 mm HU combination with GDC‐0575 had a similar level of recovery as the 0.1 mm HU combination, whereas the combination of GDC‐0575 with 0.5 μm gemcitabine resulted in a complete loss of proliferative potential (Fig. 3C,D). Two‐day HU combination treatments resulted in a higher recovery rate than 3‐day treatment. The effects of these combination treatments on checkpoint signalling and replication stress markers in the fibroblasts were examined. All treatments without GDC‐0575 resulted in CHK1 activation, indicated by the strong increase of RRM2 which was blocked by GDC‐0575 addition (Fig. 3E). Increased levels of p53 and activated phosphorylated CHK2 T68 (pCHK2), markers of ATM activation and double‐stranded DNA breaks, were observed in all treatments except 0.1 mm HU alone. Similarly, the levels of pRPA2 S33, hyperphosphorylated RPA2 and γH2AX, markers of replication stress and DNA damage, were all strongly elevated in all treatments except 0.1 mm HU alone (Fig. 3E). Surprisingly, HU + GDC‐0575 consistently increased γH2AX levels above those observed with gemcitabine with or without GDC‐0575. Thus, there was little difference between the ability of HU and gemcitabine combinations with GDC‐0575 to promote high levels of replication stress and DNA damage, with both single‐stranded and double‐stranded DNA breaks indicated by the CHK1 and CHK2 activation and p53 accumulation. The difference is in the ability of normal cells to recover from the damage and arrest. The recovered cells in the HU + GDC‐0575‐treated samples had a similar appearance to the control cells, whereas the growth‐arrested gemcitabine combination‐treated cells were enlarged with long projections, which suggests a senescent phenotype that indicates the effects of this combination result in a maintenance of DNA damage signalling (Fig. S5).

These *in vitro* data demonstrate that combination of submicromolar concentrations of GDC‐0575, within the clinically achievable concentration of this drug (Italiano *et al*., 2018), was strongly sensitised with subclinical concentrations of HU. It resulted in complete loss of cell viability in tumour cells but retention of proliferative capability in normal cells. By contrast, a low concentration of gemcitabine, well below the > 20 μm *C*_max_ levels attained in patients (Infante *et al*., 2017), resulted in complete loss of proliferative capability of normal cells as well.

### 3.3 Subclinical HU + GDC‐0575 triggers high levels of replication stress and apoptosis in melanoma and NSCLC cells

**Figure 4:**
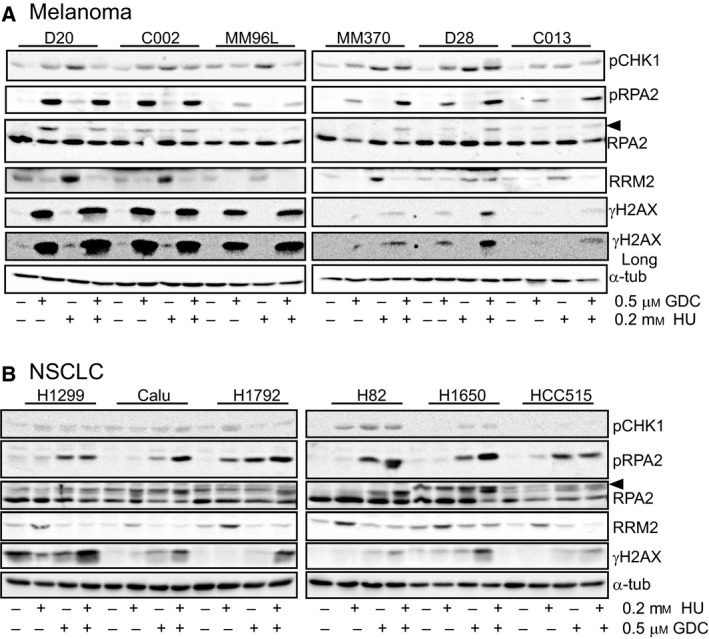
Low‐dose HU enhances replication stress in cancer cell lines. The indicated melanoma TS (A) and NSCLC cell lines (B) were treated for 24 h with the indicated combinations of 0.5 μm GDC‐0575 and 0.2 mm HU and then harvested and whole‐cell lysates immunoblotted for the indicated replication stress markers. The cell lines are in order of sensitivity to the combination, left to right, most to least sensitive. The hyperphosphorylated form of RPA2 is indicated by the arrowhead. Longer exposure of γH2AX staining demonstrates the low levels in the less sensitive TSs. α‐Tubulin was used as the loading control. These data are representative of at least two independent experiments.

A subclinical concentration of HU had little effect on DNA replication. In some melanoma cell lines, it increased the proportion of cells actively replicating their DNA, but in these cell lines, the total EdU staining intensity was reduced (Fig. S6). This reflects the S‐phase accumulation and slowdown in S‐phase progression observed with submillimolar HU observed in the NSCLC cell lines (Fig. 1A, Fig. S1A). Treatment with 0.2 mm HU increased activated pCHK1 Ser317 and CHK1 activity (demonstrated by elevated RRM2 levels) in panels of melanoma TSs and NSCLC cell lines, but had little effect on pRPA2 S4/8 and γH2AX levels (Fig. 4A,B). GDC‐0575 treatment increased both pRPA2 S4/8 and γH2AX levels in cell lines sensitive to this agent alone (D20, C002, MM96L), but the combination increased these markers in all cell lines to the greatest extent of either treatment alone (Fig. 4A,B). There was a trend for the cell lines with the lowest sensitivity to the combination treatment to also have lowest levels of pRPA2 and γH2AX. GDC‐0575 effectively reduced RRM2 levels. As reported previously (Buisson *et al*., 2015), the activation of CHK1 with the high‐dose HU appeared to be ATR‐dependent. However, using a concentration of the DNA‐PK inhibitor NU‐7441 sufficient to significantly reduce DNA‐PK‐dependent RPA2 S4/8 phosphorylation had little effect on either pCHK1 levels or the level of DNA damage indicated by γH2AX levels (Fig. S7A), which we have found to be a good marker of CHK1 inhibition (Fig. 4).

**Figure 5:**
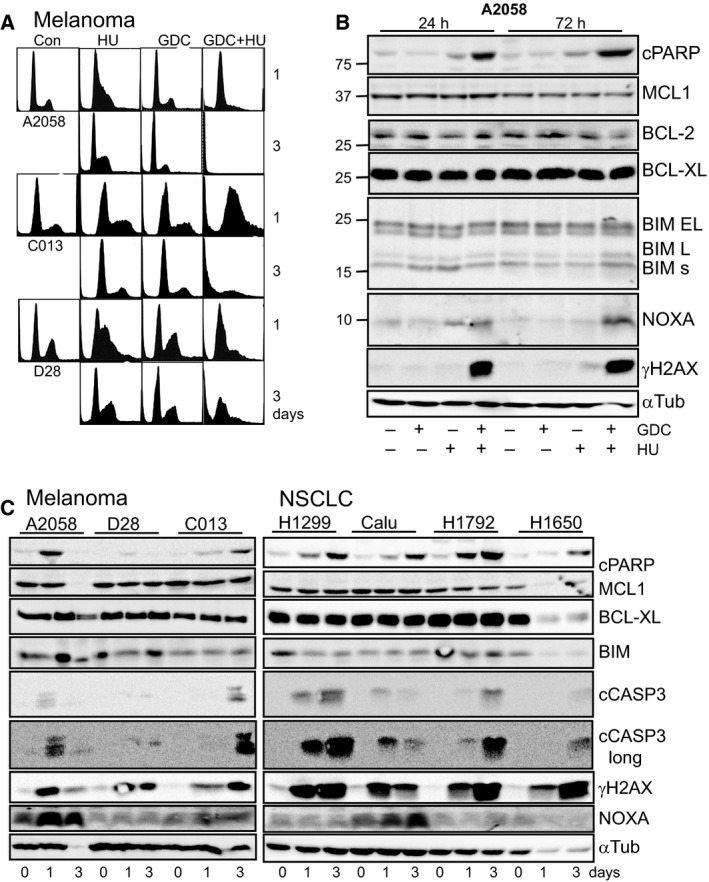
Combination of GDC‐0575 with subclinical HU promotes DNA damage and apoptosis in cancer cell lines. (A) The indicated melanoma TS lines were treated with 0.2 mm HU + 0.5 μm GDC‐0575 for 0, 1 and 3 days, then harvested and analysed by flow cytometry. (B) A2058 TSs treated as in A were immunoblotted for cleaved PARP (cPARP), the indicated BCL‐2 family proteins, the BH3‐only pro‐apoptotic proteins BIM and NOXA, and γH2AX as markers of DNA damage and apoptosis. α‐Tubulin was used as a loading control. (C) Cells from the experiment shown in A and Fig. S7B were lysed and immunoblotted for the indicated markers, including cleaved caspase‐3 (cCasp3). The data are representative of replicate experiments.

The effect of low‐dose HU on the cell cycle was to increase the proportion of cells in S phase in the first 24 h, similar to NSCLC lines (Fig. 1A, Fig. S1A) and the corroborating EdU data (Fig. S6). The combination with GDC‐0575 promoted a strong S‐phase accumulation by 24 h and loss of viability by 3 days, indicated by the increased subdiploid population (Fig. 5A, Fig. S7B). Analysis of A2058 TSs demonstrated that increased γH2AX was only observed with the combination treatment, associated with increased cleaved PARP, a marker of apoptosis, and increased levels of the BH3‐only pro‐apoptotic protein NOXA (Fig. 5B). A similar pattern was observed in three melanoma TS and four NSCLC lines. An increased subdiploid compartment correlated with increased γH2AX, cleaved caspase‐3 and its product cleaved PARP and was commonly associated with increased levels of the BH3‐only pro‐apoptotic protein NOXA (Fig. 5C). None of the other apoptosis regulatory proteins analysed showed any consistent changes. The contribution of the increased NOXA levels to the apoptosis observed was assessed by overexpressing MCL1, the selective target of NOXA (Chen *et al*., 2005), in A2058 melanoma cells. There was a small reduction in apoptosis detected by a range of markers in cells strongly overexpressing MCL1, suggesting that the NOXA–MCL1 axis is not the major regulator of apoptosis in response to the combination treatment (Fig. S8).

### 3.4 Combination of subclinical doses of HU + GDC‐0575 effectively blocks tumour growth *in vivo*

The ability of the combination of GDC‐0575 and low‐dose HU was assessed *in vivo* using xenograft models of melanoma and NSCLC. Initially, safe dosage levels of the combination were determined. We have previously reported that 50 mg·kg^−1^ GDC‐0575 was effective and well tolerated in xenograft models in immunocompromised mice (Oo *et al*., 2018). The half‐life of HU in nude mice is 11 min (Van den Berg *et al*., 1994). To maintain a dose of HU in a range where it would be effective in combination with GDC‐0575, the effect of a range of doses and schedules of HU + GDC‐0575 in nude mice with and without xenografted tumours on blood cell counts was assessed. Combination of 25 mg·kg^−1^ GDC‐0575 [half the effective single‐agent dose used previously (Oo *et al*., 2018)] with doses of HU above 150 mg·kg^−1^ was highly toxic, correlating with the collapse of white blood cell counts (Table S2). Treatment on three consecutive days was also toxic, but well tolerated on three alternate days. A final dosing schedule of 20 mg·kg^−1^ GDC‐0575 + 100 mg·kg^−1^ HU orally, followed 4 h later by 50 mg·kg^−1^ HU ip, was found to be well tolerated. There was a 30% reduction in red cell counts attributable to the combination, and a 50% reduction in white cell counts, primarily neutrophils and lymphocytes, largely attributable to HU (Table S2).

**Figure 6:**
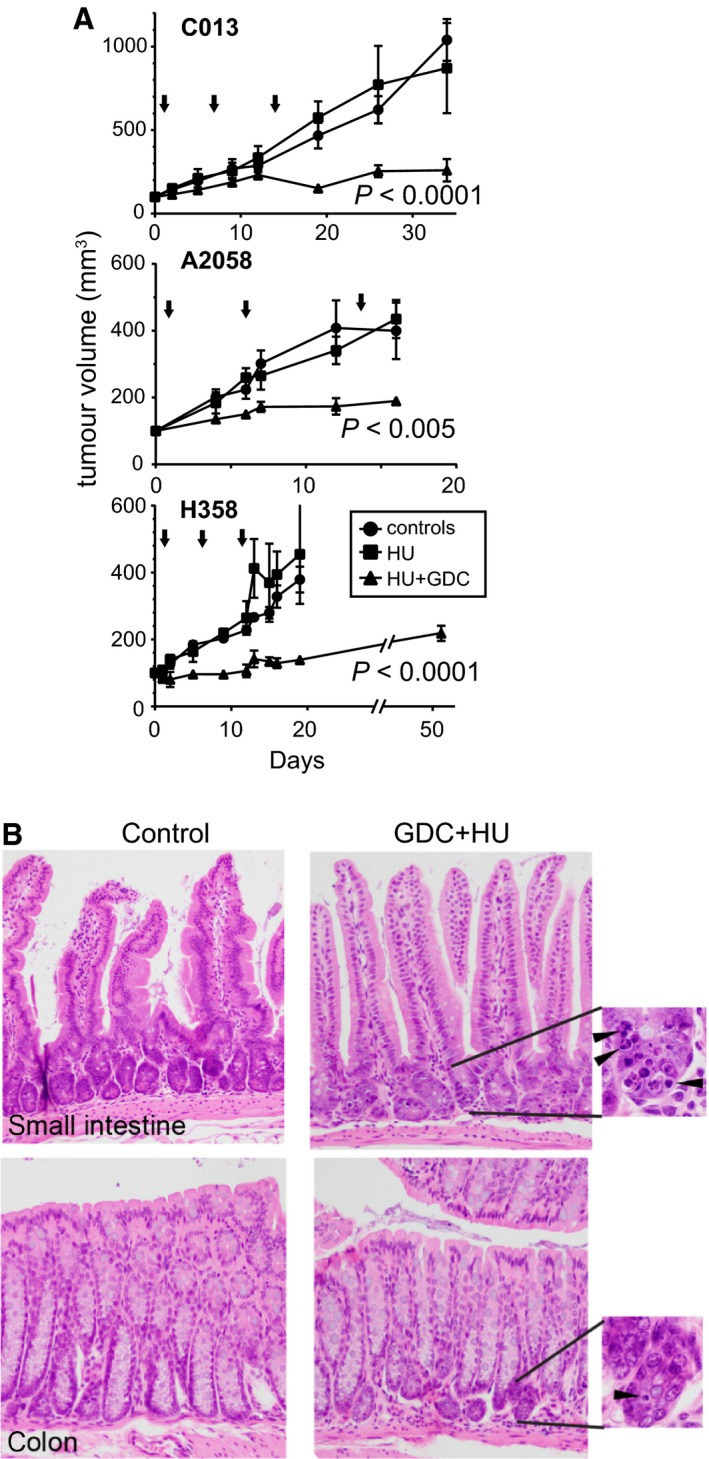
Combination of HU and GDC‐0575 controls tumour growth. (A) Melanoma (C013, A2058) and NSCLC (H358) xenografts were established in nude mice and treated with either vehicle (Con), HU or HU + GDC‐0575 using a schedule of 20 mg·kg^−1^ GDC‐0575 + 100 mg·kg^−1^ HU by oral gavage, followed 4 h later by 50 mg·kg^−1^ HU ip, only alternate days three times a week for 3 weeks. Each point represents the mean of 4–6 mice, and the error represents SD. In the case of H358, the combination‐treated mice were allowed to continue for a further 30 days after the final treatment. The arrows indicate the treatments. Significance was assessed by two‐way ANOVA for comparison of control and HU‐treated against HU + GDC‐treated samples. There was no difference between control and HU‐treated samples. (B) H&E staining of small intestines and colon from either control or combination‐treated mice killed the day after final treatment. Images were taken at 20× magnification. 60× magnification of the indicated regions in the base of the crypts shows apoptotic cells (arrowheads).

**Figure 7:**
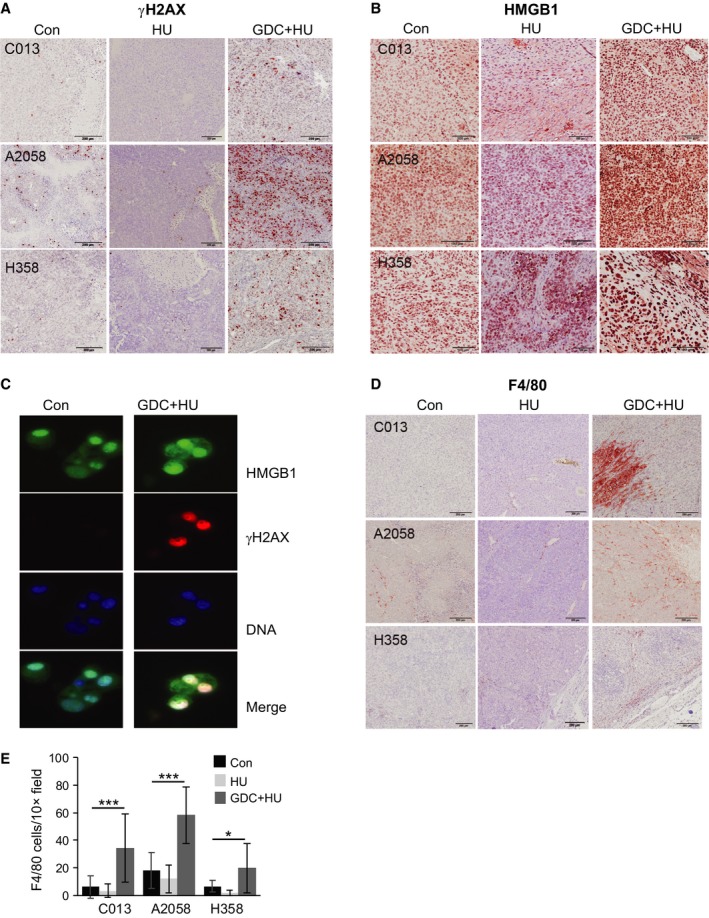
Combination of HU and GDC‐0575 promotes inflammation. Representative images of immunohistochemical staining of xenografts harvested at the end of the experiment for (A) γH2AX, (B) HMGB1 and (D) F4/80. The scale bar is 200 μm for γH2AX and F4/80 and 100 μm for HMGB1. (C) A2058 TS culture, either control (Con) or treated with 0.2 mm HU + 0.5 μm GDC‐0575 for 72 h, then fixed and stained for the indicated markers using immunofluorescence detection. (E) The number of F4/80‐stained cells (macrophages) infiltrating into the xenografts was calculated by counting five 10× fields adjacent to the edge of the tumour in three to five xenografts for each model used. The data are mean and SD for this whole population. *P < 0.05, ***P < 0.001 (Mann–Whitney *t*‐test).

Using this schedule, two melanoma and one NSCLC xenograft model were established in nude mice and treated with either HU alone or the combination HU + GDC‐0575, three treatments per week for 3 weeks. We have previously shown that only tumour cell lines hypersensitive to CHK1i *in vitro* were also sensitive to CHK1i *in vivo* as a single agent (Oo *et al*., 2018). As none of the models assessed here was hypersensitive to CHK1i, only HU alone and the combination were investigated. In each model, treatment effectively blocked tumour growth without any significant effect on mouse weight (Fig. 6A, Fig. S9A). The combination did result in apoptosis in the proliferative population at the base of intestinal crypts in the small intestines and colon, although this had no obvious effect on the architecture of the crypts (Fig. 6B), or their function as indicated by the normal mouse weights. HU‐only treatment produced a similar level of apoptosis in small intestine crypts (Fig. S9B). The combination drug treatment did not affect the proliferative capacity of any of the xenografts, measured by Ki67 staining or mitotic rate (Fig. S9C; data not shown). There was a strong increase in γH2AX staining in the combination‐treated but not HU‐only‐treated xenografts, which were harvested as much as 30 days after the final drug treatment (Fig. 7A). The increased γH2AX suggests the cells are under elevated stress, which could drive a stress response to signal extracellular factors. We observed erythema and inflammation of the skin directly over the tumours in three of the four combination‐treated A2058 xenografted mice which were not observed in any of the control or HU‐only‐treated xenografted mice. Analysis of the tumours revealed an increased intensity in nuclear staining for HMGB1 in combination‐treated tumours, with some scattered areas of increased intensity of nuclear HMGB1 staining in the HU‐only‐treated tumours (Fig. 7B). Combination drug treatment of TSs *in vitro* produced a similar increase in nuclear HMGB1 and γH2AX staining intensity and increased cytoplasmic HMGB1 staining (Fig. 7C). HMGB1 is exported out of damaged cells and acts as a cytokine for innate and adaptive immune cells such as macrophages through binding the RAGE (Cottone *et al*., 2015; Kang *et al*., 2013). We found that there was an increase in tumour‐infiltrating macrophages (F4/80 staining dendritic cells) in all of the combination‐treated but not HU‐only‐treated tumours (Fig. 7D,E). The level and pattern of macrophage infiltration varied with each tumour. A2058 xenografts consistently showed the highest level of infiltration, and this was relatively uniform in the tumour, although the macrophage numbers did decrease with tumour depth. C013 and H358 xenografts had lower numbers of infiltrating macrophages, although both tumours had regions of very high macrophage density. The variation in infiltration is indicated by the wide error bars, but in all the models used, there was a significant increase in F4/80‐stained cells infiltrating only the combination‐treated tumours.

## 4 Discussion

The clinical limitation on the combination of CHK1i with gemcitabine is the toxicity in normal tissues, commonly haematological (Daud *et al*., 2015; Infante *et al*., 2017; Italiano *et al*., 2018). Here, we have investigated another well‐known replication stress promoter, HU, and found that subclinical concentrations effectively sensitise a broad range of melanoma and NSCLC lines to clinically achievable doses of the CHK1i GDC‐0575 and GNE‐323. The surprising feature was that combination of HU with CHK1i was less toxic to normal tissue *in vivo* and *in vitro*. Normal fibroblasts retained proliferative potential after extended treatment with even high concentrations of HU + GDC‐0575, whereas an equivalent concentration of gemcitabine + GDC‐0575 produced a complete loss of proliferative potential. The mechanism of these two RNR inhibitors is likely to underlie the difference in the effect of the combination with CHK1i in normal tissue.

Gemcitabine was found to produce a complete S‐phase checkpoint arrest and maximal checkpoint activation at concentrations as low as 50 nm. Lower doses (10–20 nm) have been reported to produce an S‐phase accumulation similar to that produced with < 0.2 mm HU (Karnitz *et al*., 2005; Matthews *et al*., 2007). Inhibition of ATR is also shown to strongly synergise with these low doses of gemcitabine (Huntoon *et al*., 2013; Prevo *et al*., 2012; Wallez *et al*., 2018), whereas we found that only higher concentrations (> 1 mm) of HU sensitised melanoma TSs to ATR inhibition. We have shown that low concentrations of gemcitabine (down to 50 nm) activate ATR–CHK1 signalling, consistent with the ability of ATR inhibitor to synergise with even lower doses of gemcitabine. By contrast, low concentrations of HU activate CHK1, although there is only modest ATR activation, and inhibition of ATR only weakly synergised with low‐dose HU compared to 2 mm HU. The role for DNA‐PK is unclear. However, the mechanism by which CHK1 is activated does not appear to influence either tumour or normal tissue sensitivity to the CHK1i, as 0.1–0.2 mm HU effectively sensitised tumour cells to the CHK1i, whereas normal fibroblasts retained similar levels of proliferative potential after CHK1i treatment with either 0.1 or 2 mm HU. This contrasted with the complete loss of proliferative potential with gemcitabine + GDC‐0575 combination. In vivo, low‐dose HU + GDC‐0575 had a modest effect on white cell counts and rapidly proliferating cells in the intestinal crypt, indicating that this combination is well tolerated by even rapidly proliferating normal tissue. One explanation for the difference in normal tissue sensitivity to the combination of CHK1i with gemcitabine or HU is the readily reversible nature of HU, whereas gemcitabine produces longer lasting effects (Montano *et al*., 2013, 2017). This is likely to be a direct consequence of the difference in their mechanisms of action. Gemcitabine (20,20‐difluoro‐20‐deoxycytidine; dFdC) is a prodrug analogue of deoxycytidine. The active di (dFdCDP)‐ and triphosphate metabolites (dFdCTP) can either covalently bind and inhibit the RRM1 subunit of RNR to reduce dNTP pools (dFdCDP) or be incorporated into the replicating DNA resulting in chain termination (dFdCTP), its primary mode of action (de Sousa Cavalcante and Monteiro, 2014). By contrast, HU is a readily reversible inhibitor of the catalytic RRM2 subunit of RNR (Gwilt and Tracewell, 1998). There was little difference in the immediate effects of these combinations on the level of DNA damage or responses to the damage. However, the enlarged, flattened morphology of gemcitabine combination‐treated fibroblasts indicates they were likely to have undergone senescence, suggesting that the DNA damage incurred with the combination treatment could not be repaired and the prolonged DNA damage response promoted senescence. The inhibition of CHK1 would overcome the S‐phase checkpoint arrest, and this might be expected to increase the incorporation of the chain terminating dFcCTP into the replicating DNA, reducing the likelihood of successful repair. In the case of HU‐induced replication stress, the reversible nature of the arrest would allow those cells where excessive levels of replication fork collapse have not occurred, to repair and recommence proliferating.

The major difference between tumour and normal cells was the reversibility of the antiproliferative effects of the low‐dose HU + GDC‐0575 combination in normal cells and complete loss of proliferative capacity in the treated tumour cells. This is likely to reflect the elevated level of endogenous replication stress in both melanomas and NSCLCs, and the relative ability of the tumour cells to repair the DNA damage incurred. The relatively poor repair capacity of the tumour cells was demonstrated by the elevated level of γH2AX staining observed in the xenografts weeks after the final treatment, which was not detected in the associated stromal cells. The ability of the NFFs to reproliferate after removal of the combination treatment is further evidence of this difference in the ability of tumour and normal cells to recover from the elevated replication stress imposed by the drug combination. *In vivo*, the apparently normal architecture and function of GI tract despite the presence of apoptotic cells in proliferating cells in the base of intestinal crypts in treated animals is evidence that normal tissues can tolerate the combination treatment.

The other interesting finding was that the CHK1i + HU combination treatment triggered an inflammatory response in the xenograft models tested here. DNA damage induced by the combination of HU and CHK1i is well known and regulated by MRE11‐MUS81 nucleases (Techer *et al*., 2016; Thompson *et al*., 2012). In prostate cancer cells, MUS81 is responsible for cytoplasmic single‐stranded DNA (ssDNA) that is detected by the cGAS‐STING pathway, and is responsible for interferon induction that can recruit macrophages to control tumour growth (Ho *et al*., 2016). HMGB1 can also bind ssDNA extracellularly to enhance binding and activation of TLR9 and RAGE‐dependent cytokine production (Bianchi, 2009), and intracellularly bend long DNA fragments to enhance activation of cGAS (Andreeva *et al*., 2017). Thus, by releasing the inhibition of MUS81, CHK1i is likely to increase the level of cytoplasmic ssDNA and cGAS–STING pathway‐dependent cytokine production. This indicates that the combination of CHK1i + low‐dose HU can trigger an inflammatory response that recruits components of the innate immune system to control tumour growth. The extent of the response does appear to vary from tumour to tumour, although a response was observed in all three xenograft models used here, suggesting there are multiple factors influencing the level of the response. Identification of these factors will be important to enhancing the ability of the immune system to productively engage the tumour. The low‐dose HU combination is well tolerated by immune effectors which should facilitate an immune response to the tumour.

## 5 Conclusion

We have found that subclinical doses of HU sensitise a high proportion of melanoma and NSCLC cell lines to clinically achievable doses of the CHK1i GDC‐0575. Normal cells tolerated the same treatment and demonstrated they retained proliferative potential. By contrast, low‐concentration gemcitabine combination with GDC‐0575, although previously shown to be effective against a range of tumour cell lines (Xiao *et al*., 2013), also resulted in a complete loss of proliferative potential in normal cells. HU + GDC‐0575 treatment provoked an inflammatory response in the xenografts that resulted in increased tumour‐infiltrating macrophages. Combined, these data indicate that the combination of CHK1i with low‐dose HU is superior to the currently tested combinations with gemcitabine due to its better tolerability and potential to trigger productive immune responses.

## Conflict of interest

The authors declare no conflict of interest.

## Author contributions

ZYO, MP, AJS, VB, CS, JEL and BG designed the experiments. ZYO, MP, AJS, DN, MF, SMD, CL, SW, VB and BG performed the experiments presented. ZYO, MP, AJS, DN, MF, SMD, SB, VB, DS, and BG analysed the data and prepared figures for the manuscript. CS performed the pathology analysis of the xenografts. All authors contributed to writing and editing this manuscript.

## Supporting information


**Fig. S1.** (A) The NSCLC cell line Calu‐1 was treated with the indicated concentrations of HU or Gemcitabine (Gem) for 24 h and then harvested and analysed for S‐phase arrest using EdU incorporation measured by high‐content imaging. (B) Samples from Calu‐1 cells treated as in A were lysed and immunoblotted for the indicated markers of replication stress. The hyperphosphorylated form of RPA2 is indicated by the arrowhead. α‐Tubulin is the loading control.
**Fig. S2.** (A) Calu‐1 cells were treated either with or without the indicated dose of HU + 0.5 μm GDC‐0575 for 48 h, the drug was washed off, and 300 viable cells were plated in three replicate wells of a 6‐well plate. Cells were allowed to grow for a further 10 days until colonies were visible, then fixed and stained with crystal violet. (B) Similar experiment to A except the indicated melanoma tumour sphere lines were treated with or without 0.2 mm HU + 0.5 μm GDC‐0575 for 72 h. Tumour spheres were dissociated into individual cells, and 300 viable cells were plated in three replicate wells of a 6 well plate as above and grown as attached cells in 10% serum. Colony formation was assessed as in A.
**Fig. S3.** The sensitivity of indicated melanoma lines to GNE‐323, a closely related CHEK1 inhibitor (Oo *et al*., 2018) or the ATR inhibitor VE‐821, with or without 0.2 mm HU for 72 h.
**Fig. S4.** The sensitivity of indicated NSCLC cell lines to GDC‐0575, with or without 0.2 mm HU for 72 h.
**Fig. S5.** Microscopy images of crystal violet‐stained NFF cells from the experiment shown in Fig. 3C.
**Fig. S6.** The percentage of EdU‐positive cells (%EdU+) and intensity of EdU staining in the indicated cell lines.
**Fig. S7.** (A) The indicated NSCLC cell lines were treated with or without the indicated concentration of HU and 0.5 μm ATR inhibitor VE‐821 (ATRi) or 2 μm NU7441 (DNAPKi) for 24 h, the cells lysed and immunoblotted for the indicated markers of CHK1 activation, replication stress, and DNA damage. The hyperphosphorylated form of RPA2 is indicated by the arrowhead. (B) The indicated NSCLC cell lines were treated with or without 0.2 mm HU, 0.5 μm GDC‐0575 or the combination for 1 or 3 days. Cells were harvested and analysed by flow cytometry for their DNA content.
**Fig. S8.** (A) A2058 melanoma cells transduced with either empty vector (Vec) or MCL1 expressing lentivirus were treated with and without 0.2 mm HU + GDC‐0575 for 48 h then lysates immunoblotted for the indicated proteins. α‐Tubulin is the loading control. (B) The same cell lines treated as in A were stained with Annexin V–FITC and propidium iodide (PI). These data are representative of two replicate experiments.
**Fig. S9.** (A) Mouse weights for the xenograft experiments shown in Fig. 6A. Error bars represent SD. (B) H&E staining of small intestines from either control, HU‐only‐treated or combination‐treated mice killed the day after final treatment. Images were taken at 20× magnification. 60× magnification of the indicated regions in the base of the crypts shows apoptotic cells (arrowheads). (C) A2058 xenograft from either control or GDC‐0575 + HU xenografts harvested at the end of the experiment were immunostained for Ki67. This is representative of at least three separate xenografted tumours for each of the three different tumours tested.
**Table S1.** IC_50_ values for the Melanoma (A) and NSCLC cancer lines (B) calculated from the data presented in Fig. 2A and Fig. S4. Sensitisation is the ratio of IC_50_ values for CHK1i alone/CHK1i + HU treatment.
**Table S2.** Blood counts of treated mice.
