# Supplementary material for: Combined use of subclinical hydroxyurea and CHK1 inhibitor effectively controls melanoma and lung cancer progression, with reduced normal tissue toxicity compared to gemcitabine
Source: Mol Oncol. 2019 Jun 14;13(7):1503–18. doi: 10.1002/1878-0261.12497 (PMC6599846; doi:10.1002/1878-0261.12497)

Supplementary Material

Supplementary Figure Legends

Supplementary Figure S1

A. The NSCLC cell line Calu-1 was treated with the indicated concentrations of HU or Gemcitabine (Gem) for 24 h then harvested and analysed for S phase arrest using EdU incorporation measured by high content imaging. The data are the mean of triplicate experiments and the error bars represent SD. **B**. Samples from Calu-1 cells treated as in A were lysed and immunoblotted for the indicated markers of replication stress. The hyperphosphorylated form of RPA2 is indicated by the arrowhead. α−Tubulin is the loading control.

Supplementary Figure S2

A. Calu-1 cells were either treated with or without the indicated dose of HU + 0.5 μM GDC-0575 for 48 h, the drug was washed off and 300 viable cells were plated in three replicate wells of a 6 well plate. Cells were allowed to grow for a further 10 days until colonies were visible, then fixed and stained with crystal violet. **B.** Similar experiment to A except the indicated melanoma tumour sphere lines were treated with or without 0.2 mM HU+0.5 μM GDC-0575 for 72 h. Tumour spheres were dissociated into individual cells, and 300 viable cells were plated in three replicate wells of a 6 well plate as above and grown as attached cells in 10% serum. Colony formation was assessed as in A.

Supplementary Figure S3

The sensitivity of indicated melanoma lines to GNE-323, a closely related CHEK1inhibitor (1) or the ATR inhibitor VE-821, with or without 0.2 mM HU for 72 h. The data are the mean of four experiments and the error bars represent SD.

Supplementary Figure S4

The sensitivity of indicated NSCLC cell lines to GDC-0575, with or without 0.2 mM HU for 72 h. The data are the mean of four replicates and the error bars represent SD.

Supplementary Figure S5

Microscopy images of Crystal violet stained NFF cells from the experiment shown in Figure 3C. Scale bars are 100 μm.

Supplementary Figure S6

The percentage of EdU positive cells (%EdU+) and intensity of EdU staining in the indicated cell lines. The data are the mean of four replicates and the error bars represent SD.

Supplementary Figure S7

A. The indicated NSCLC cell lines were treated with or without the indicated concentration of HU and 0.5 μM ATR inhibitor VE-821 (ATRi) or 2 μM NU7441 (DNAPKi) for 24 h, the cells lysed and immunoblotted for the indicated markers of CHK1 activation, replication

stress, and DNA damage. The hyperphosphorylated form of RPA2 is indicated by the arrowhead. **B.** The indicated NSCLC cell lines were treated with or without 0.2 mM HU, 0.5 μM GDC-0575 or the combination for one or three days. Cells were harvested and analysed by flow cytometry for their DNA content.

Supplementary Figure S8

A. A2058 melanoma cells transduced with either empty vector (Vec) or ~~MCL-1~~ expressing lentivirus were treated with and without 0.2 mM HU+GDC-0575 for 48 h then lysates immunoblotted for the indicated proteins. α−Tubulin is the loading control. **B.** The same cell lines treated as in A were stained with Annexin V-FITC and propidium iodide (PI). These data are representative of two replicate experiments.

Supplementary Figure S9

A. Mouse weights for the xenograft experiments shown in Figure 6A. The data are the mean and SD of at least five mice. **B**. H&E staining of small intestines from either control, HU only or combination treated mice sacrificed the day after final treatment. Images were taken at 20x magnification. 60x magnification of the indicated regions in the base of the crypts shows apoptotic cells (arrowheads). **C**. A2058 xenograft from either control or GDC-0575+HU xenografts harvested at the end of the experiment were immunostained for Ki67. This is representative of at least three separate xenografted tumours for each of the three different tumours tested. Scale bars are 100 μm.

Supplementary Table S1:

A

	IC_{50} GDC-0575 (nM)	
	**CHK1i**	**CHK1i+HU**
D20	94	22
C045	155	66
C002	183	27
A15	216	29
MM96L	216	70
D04	226	3
HT144	278	15
C052	410	81
A2058	1180	3
C054	1380	3
SKMEL13	1439	1
MM370	1694	132
MM603	2178	3
D28	3048	55
BL	3767	240
MM415	3819	1
MM329	3837	129
C013	10000	54
SKMEL28	10000	152
D25	30000	195

B

	IC_{50} GDC-0575 (nM)	
	**CHK1i**	**CHK1i+HU**
HCC4017	110	10
H1299	212	10
HCC2429	274	17
H2052	315	10
Calu-1	319	10
H1792	352	10
H358	478	10
H82	524	44
H322	594	70
H1975	832	32
H1650	4236	344
H2887	7194	33
HCC515	30000	30000

IC50 values for the Melanoma (A) and NSCLC cancer lines (B) calculated from the data presented in Figure 2A and Supplementary Figure S4.

Supplementary Table S2: Blood counts of treated mice

Schedule	Treatment		RBC 10e12/L	Hgb g/L	WBC x10e9/L	Neutr# x10e9/L	Lym# x10e9/L	Mono# x10e9/L	Eos# x10e9/L	
No tumour 3 consecutive days treatment	25mg/kg GDC+500mg/kg HU po then 250mg/kg HU po	ut1	10.52	167	12.95	2	10.2	0.49	0.24	
		ut2	10.3	174	14.66	2.52	11.27	0.74	0.14	
		3CD#0	6.56	108	0.88	0.04	0.48	0.34	0.02	**Highly toxic**
		3CD#1	11	184	0.5	0.2	0.24	0.07	0.07	**Highly toxic**
No tumour alternative days treatment	25mg/kg GDC +250mg/kg HU po then 150mg/kg HU po	ut	8.82	146	7.32	1.96	5.04	0.2	0.12	
		AD-#0	7.3	120	0.5	0.08	0.4	0	0.02	**Highly toxic**
		AD#1	9.3	154	0.66	0	0.62	0.04	0	**Highly toxic**
No tumour	25mg/kg GDC + 150mg/kg HU po then 75mg/kg po	ut	10.52	162	6.92	1.54	4.74	0.4	0.22	
		CD#0	7.84	128	0.14	0.04	0.08	0.02	0	**Highly toxic**
		AD#0	9.3	156	3.08	1.06	2.02	0	0	**Tolerated**
		AD#1	8.68	148	1.24	0.36	0.86	0.02	0	**Tolerated**
C002 Xenografts alternative days treatment	20mg/kg GDC +100mg/kg HU po then 50mg/kg HU ip	ut	12.68	192	19.34	12.18	5.72	1.16	0.24	
		comb	6.1	94	7.72	3.6	3.44	0.48	0.16	**Well Tolerated**
		comb	11.22	158	4.32	1.9	2.04	0.24	0.1	**Well Tolerated**
A2058 Xenografts 3 alternate days treatment for 3 cycles	20mg/kg GNE + 100mg/kg HU po then 4 hrs later 50mg/kg HU ip on 3 alternate days for 3 cycles	veh1-1	8.78	131	4.54	1.94	2.32	0.19	0.09	
		veh1-2	10.08	152	5.1	1.44	3.42	0.1	0.14	
		HU2-1	9.06	147	3.07	0.68	2.19	0.14	0.06	
		HU2-2	8.93	138	1.53	0.58	0.86	0.06	0.03	
		comb4-1	6.19	98	2.12	0.69	1.14	0.18	0.1	**Well Tolerated**
		comb4-2	6.12	98	2.41	0.68	1.56	0.09	0.07	**Well Tolerated**
		comb4-3	4.52	73	1.31	0.49	0.6	0.14	0.08	**Well Tolerated**
		comb4-4	7.23	112	1.8	0.43	1.22	0.09	0.06	**Well Tolerated**

Supplementary Figure S1

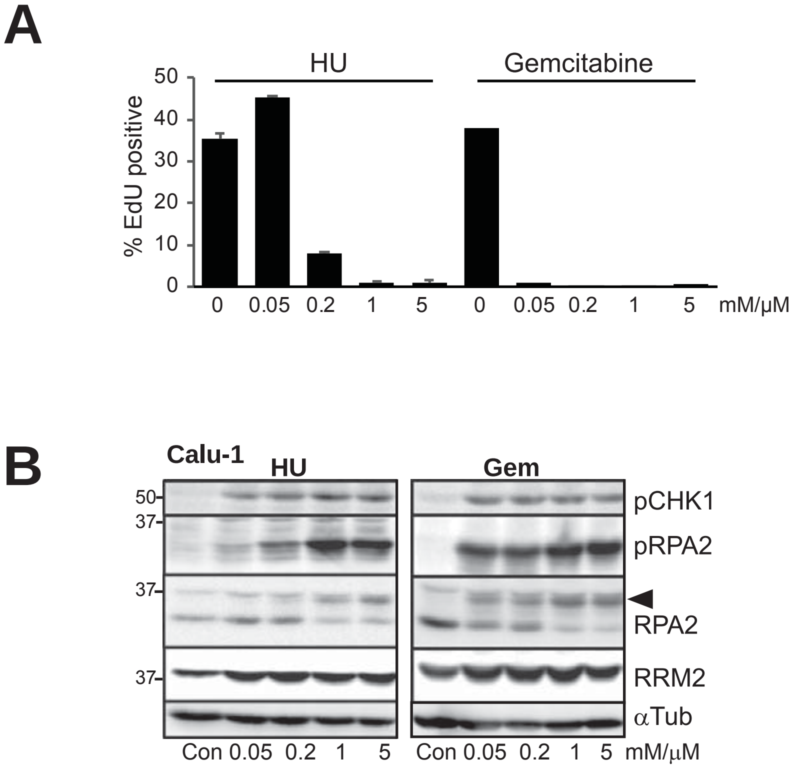

Supplementary Figure S2

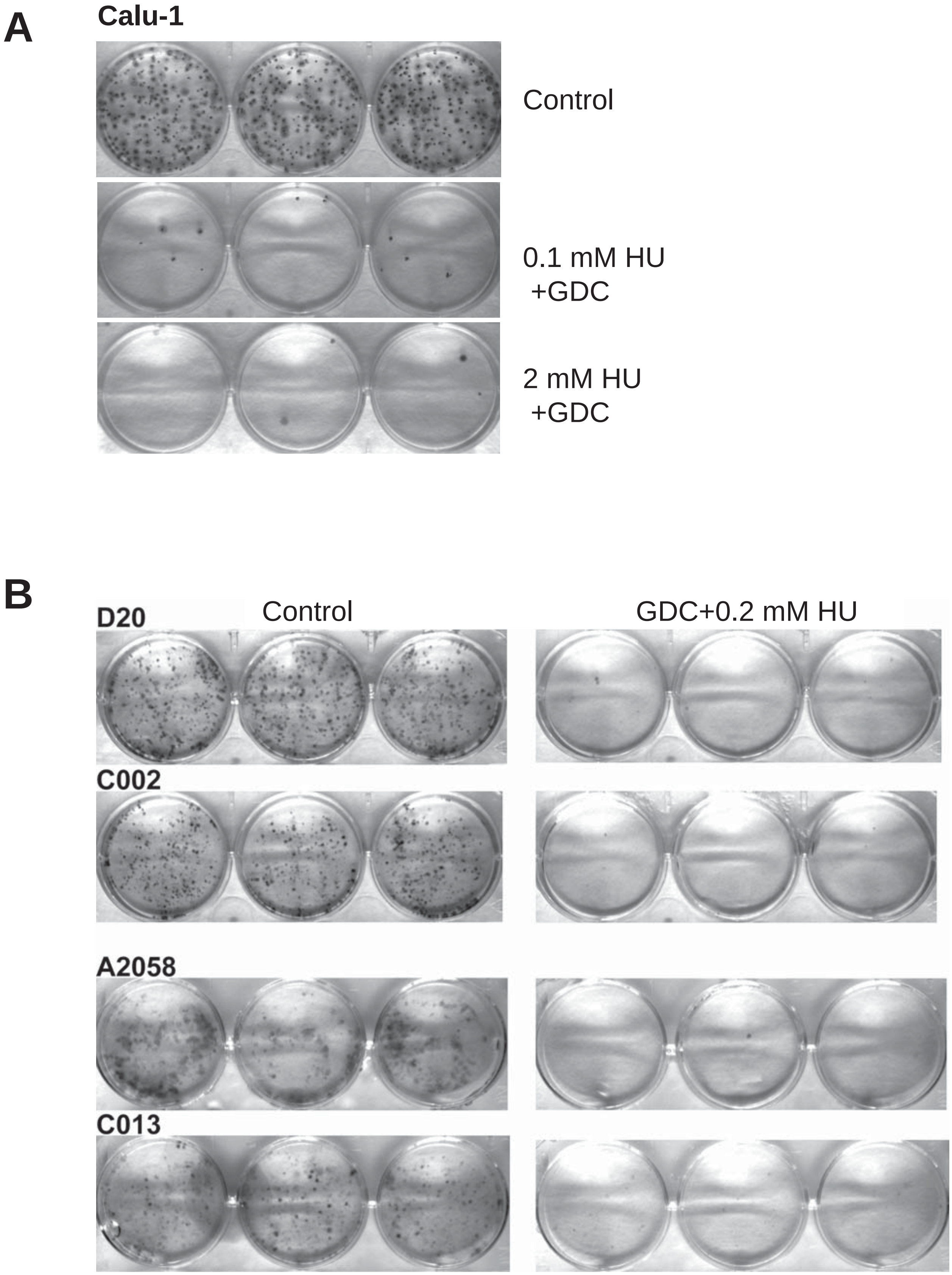

Supplementary Figure S3

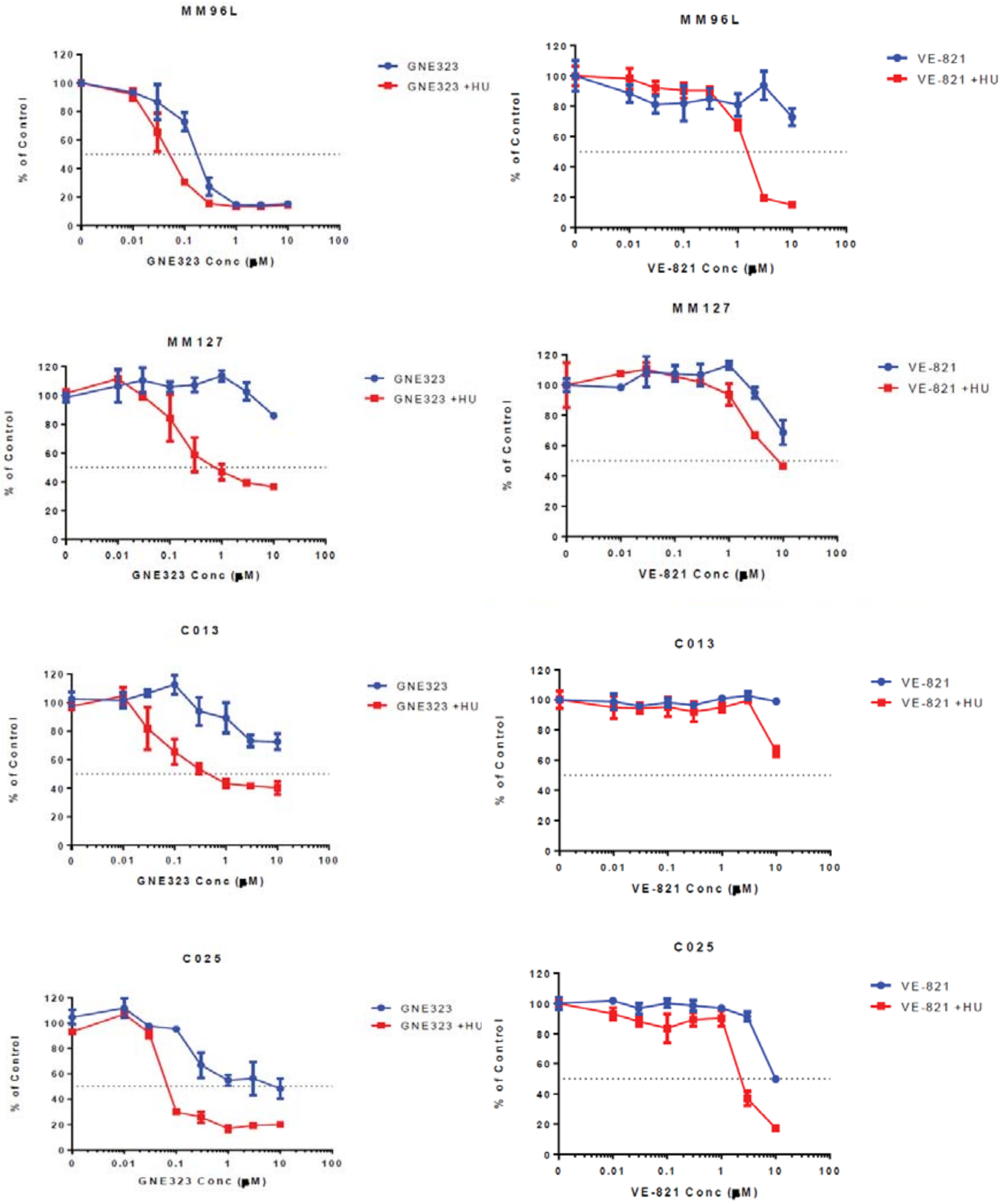

Supplementary Figure S4

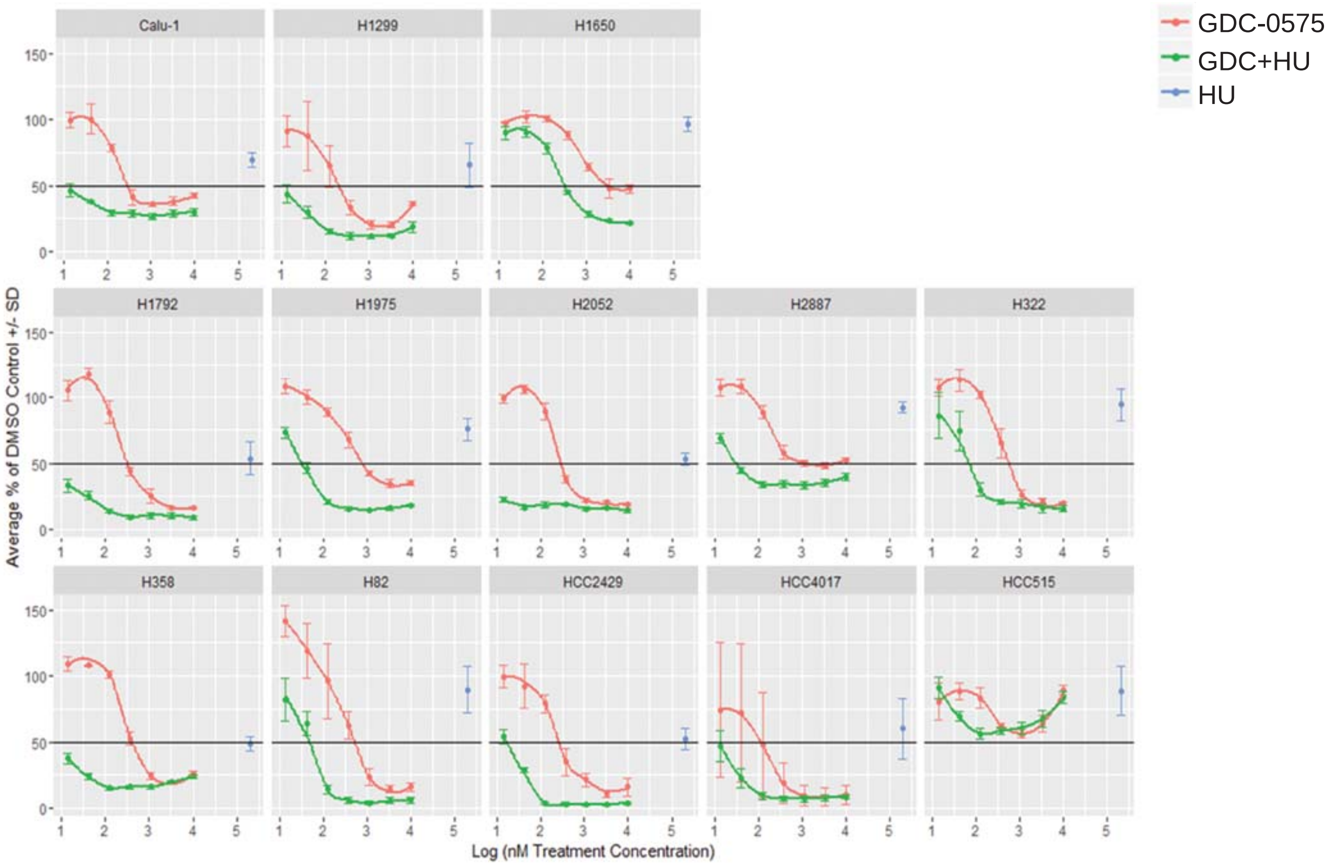

Supplementary Figure S5

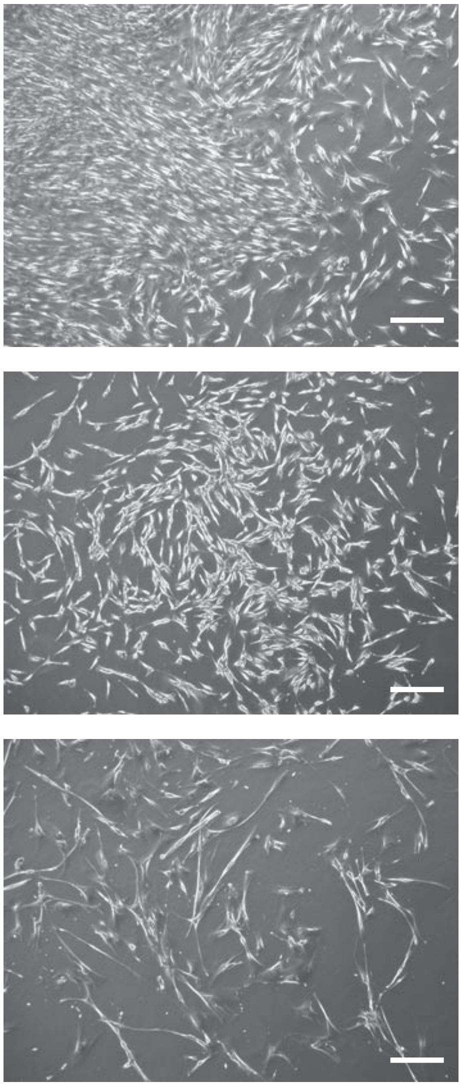

Control

0.1 mM HU
+GDC

0.5 μM Gem
+GDC

Supplementary Figure S6

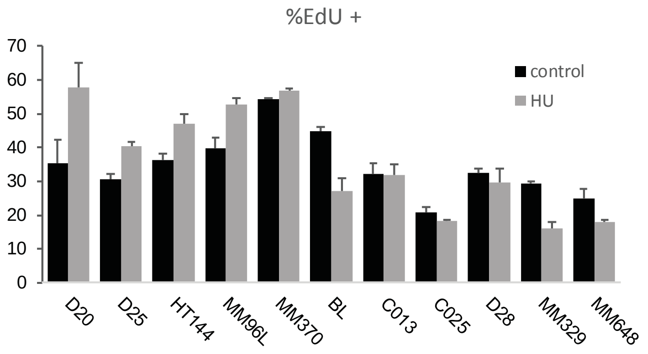

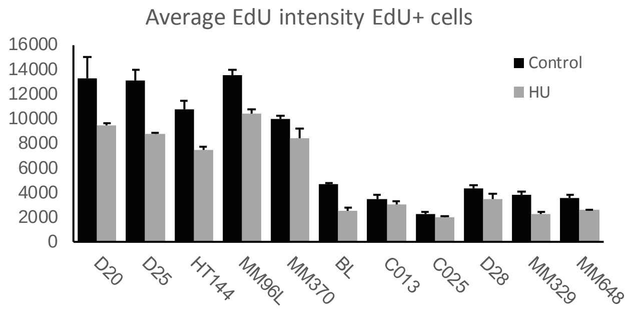

Supplementary Figure S7

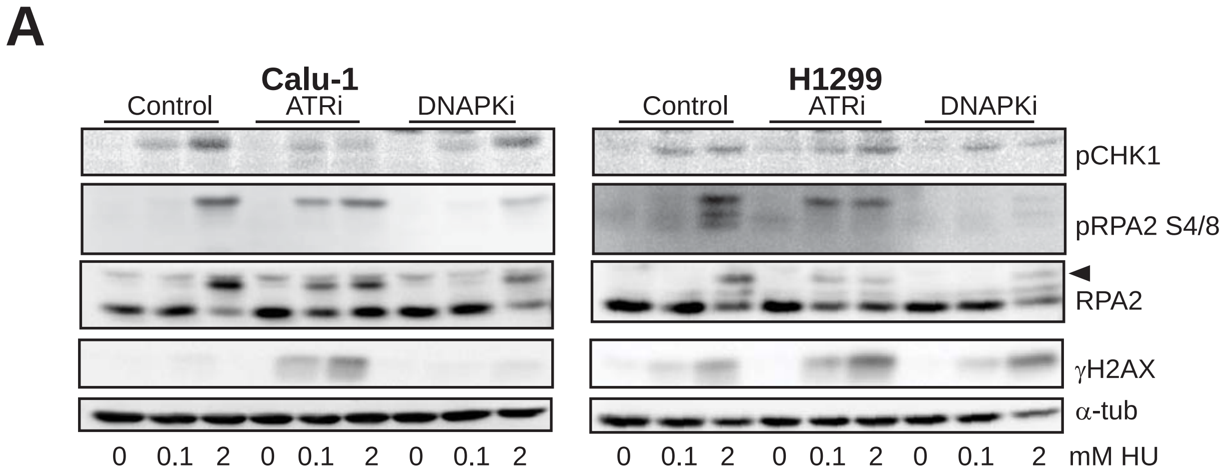

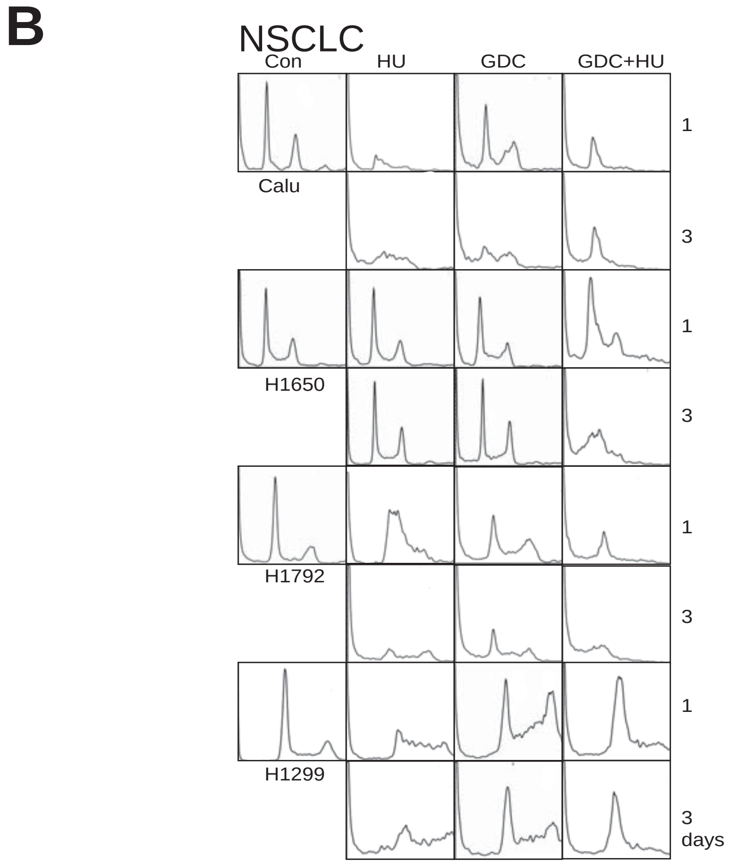

Supplementary Figure S8

A

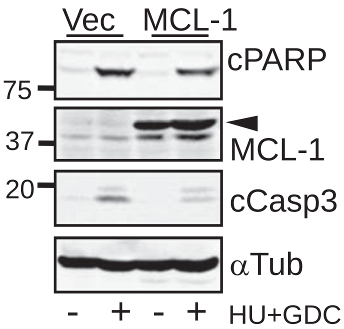

B

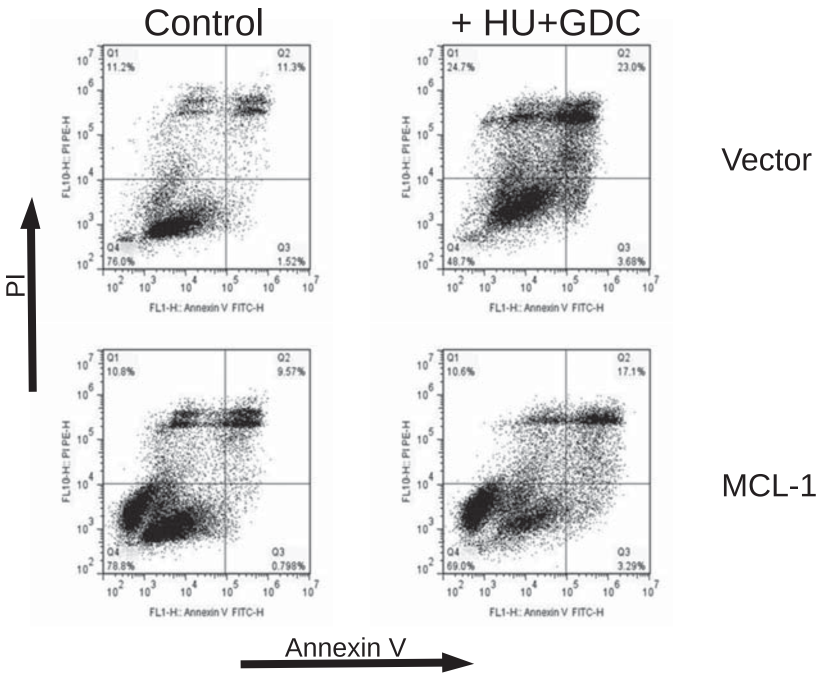

Supplementary Figure S9

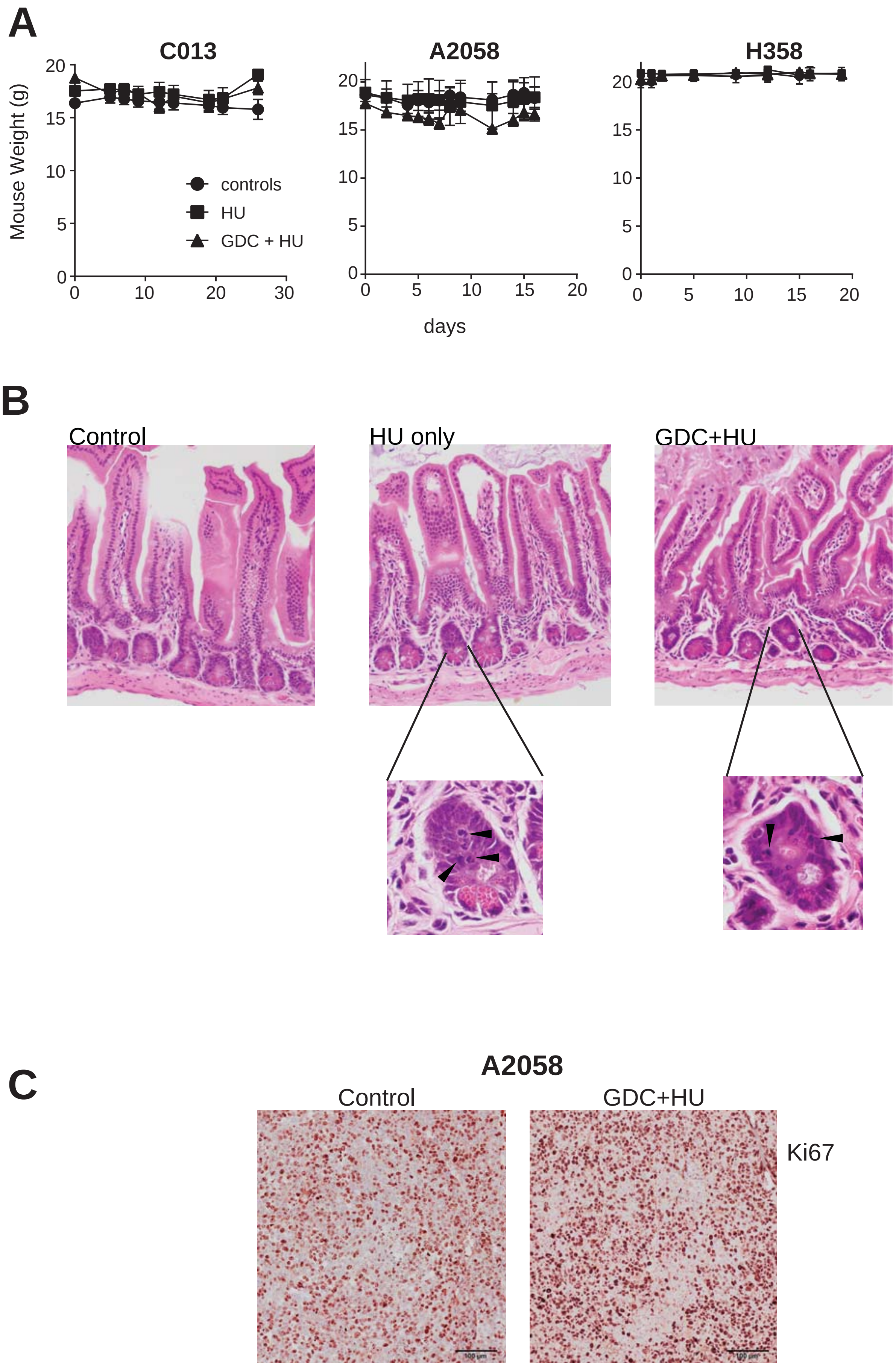

Supplement: Supplementary file 1 — Fig. S1. (A) The NSCLC cell line Calu‐1 was treated with the indicated concentrations of HU or Gemcitabine (Gem) for 24 h and then harvested and analysed for S‐phase arrest using EdU incorporation measured by high‐content imaging. (B) Samples from Calu‐1 cells treated as in A were lysed and immunoblotted for the indicated markers of replication stress. The hyperphosphorylated form of RPA2 is indicated by the arrowhead. α‐Tubulin is the loading control. Fig. S2. (A) Calu‐1 cells were treated either with or without the indicated dose of HU + 0.5 μm GDC‐0575 for 48 h, the drug was washed off, and 300 viable cells were plated in three replicate wells of a 6‐well plate. Cells were allowed to grow for a further 10 days until colonies were visible, then fixed and stained with crystal violet. (B) Similar experiment to A except the indicated melanoma tumour sphere lines were treated with or without 0.2 mm HU + 0.5 μm GDC‐0575 for 72 h. Tumour spheres were dissociated into individual cells, and 300 viable cells were plated in three replicate wells of a 6 well plate as above and grown as attached cells in 10% serum. Colony formation was assessed as in A. Fig. S3. The sensitivity of indicated melanoma lines to GNE‐323, a closely related CHEK1 inhibitor (Oo et al., 2018) or the ATR inhibitor VE‐821, with or without 0.2 mm HU for 72 h. Fig. S4. The sensitivity of indicated NSCLC cell lines to GDC‐0575, with or without 0.2 mm HU for 72 h. Fig. S5. Microscopy images of crystal violet‐stained NFF cells from the experiment shown in Fig. 3C. Fig. S6. The percentage of EdU‐positive cells (%EdU+) and intensity of EdU staining in the indicated cell lines. Fig. S7. (A) The indicated NSCLC cell lines were treated with or without the indicated concentration of HU and 0.5 μm ATR inhibitor VE‐821 (ATRi) or 2 μm NU7441 (DNAPKi) for 24 h, the cells lysed and immunoblotted for the indicated markers of CHK1 activation, replication stress, and DNA damage. The hyperphosphorylated form of RPA2 is in [file MOL2-13-1503-s001.pdf]
